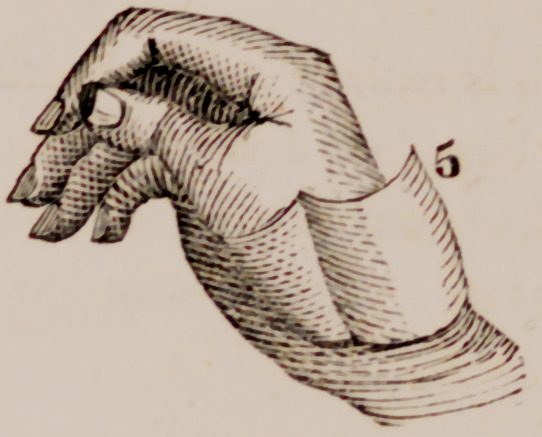# Rhinoplasty, Blepharoplasty and Cheiloplasty in the Same Patient

**Published:** 1849-01

**Authors:** F. H. Hamilton


### BUFFALO MEDICAL JOURNAL

AND

### MONTHLY REVIEW.

**VOL. 4.** JANUARY, 1849. **NO. 8.**

### ORIGINAL COMMUNICATIONS.

ART I.—*Rhinoplasty, Blepharoplasty and Cheiloplasty in the same Patient.* By Dr. F. H. Hamilton.

B.—Aged about 25. When a child Mr. B, fell into the fire and was so terribly burned that he lost a great portion of his nose; his left eyelids became deformed by an extensive retraction and eversion of the upper lid, and by a similar retraction and eversion of the lower lid; the left angle of his mouth was closed to the extent of nearly an inch, and the whole mouth drawn to the left side; the left side of the face became throughout a solid and irregular cicatrix.

This was his condition when he first presented himself to me in March, 1847. My *first* operation, made in presence of the medical class of the

University, was upon the left upper lid. A triangular shaped piece was cut from the lid, the base of which corresponded with the tarsal border; and above this, along the line of the superciliary ridge an incision was made of about eight lines in length, (1) from which the dissection was continued downward, until the upward retraction of the lid was entirely relieved. The triangular wound on the tarsus was now closed by two delicate sutures.

The *second* operation was made at the same time upon the lower lid; and in the same manner.

The *third* operation was made a few days later, in presence of the class also, upon the mouth. The mouth being much drawn to the left, the right commisure was made to approach near the centre of the jaw; while the left commisure was also advanced toward the mesian line by adhesion; the contraction of the cicatrix had also lifted it up five or six lines above the opposite commisure. I first made an incision upon the left angle, carrying the knife a little *downward* as well as outward, to the extent of about eight lines. The same operation was immediately made upon the right angle, the knife however being here carried a little *upward* and outward about six lines. Both of these incisions were commenced from within, the mucous membrane being first incised about four lines above the commisure. and from thence dissected down to the commisure, after which the muscle and integument were divided together (3–3). The flaps of mucous membrane were now brought over and secured to the tegumentary edge of the wound by three light sutures on each side.

As soon as the incision upon the lids and mouth had healed, and the operation upon the lower lid was found to be uusatisfactory, the *fourth* operation was made. The eversion and retraction were diminished, but owing to the complete inelasticity of the integuments below, the improvement was not such as was desired. I therefore made a horizontal incision below the lid and then dissected upward to the border of the tarsus. A flap was now raised from the lower part of the temple, the pedicle of which reached the outer extremity of the incision just made, and by tortion this flap was placed in the wound below the lid, and secured by sutures and compress.

These operations having been completed, the patient went home to re-establish his health, which had suffered some from his confinement and the constant irritations of successive operations.

In the spring of 1848 he returned and on the 11th of March, in presence of ten or fifteen of the medical students and several physicians, I prepared to restore the right side of his nose. This, the *fifth* operation, consisted of two distinct stages. The right side of the face being unscarred and healthy, I determined to make here the operation of "sliding" (*par glissement du lambeau*). The vestige of the nose was first made raw upon this side; two parallel incisions were then made outward (4), one commencing at the point where the ala of the nose should terminate below, and extending outward about two inches, care being taken to avoid the duct of Steno; the other commencing about an inch higher up, and reaching outward the same distance. Both incisions were deep. The intercepted piece was now dissected up from the nose outward to the extent of the tegumentary incisions. The infra orbital artery required a ligature. Hæmorrhage having ceased, the flap was brought forward and with sutures, adhesive straps and compresses secured in place upon the side and top of the right nostril.

The second stage of this operation consisted in separating, two weeks later, the flap thus raised, from its base so as to allow the cheek to fall back to its place, and at the same time relieve the strain upon the nose. The incision was made a little convex outward and at a point corresponding to the position of the base of the ala, so as to leave a depression resembling the natural depression at this point.

Erysipelas having twice during these last operations attacked his face, we did not attempt the restoration of the opposite side until the last of March.

*Sixth* operation. This was divided into three separate stages. No integument could be taken from the left side of the face, and I was unwilling to make a new scar by operating from the forehead. Having already tested the courage and endurance of my patient, I concluded to make the flap from the right hand.

March 29th I dissected from the ball of the thumb of the right hand a piece of integument of a quadrilateral form, (5), two inches in length by one and a half in breadth, its longest diameter extending from within outwards and its base or point of attachment resting over the radial border of the metacarpal bone of the index finger. The palmar portion of this flap was at this time covered with a heavy cuticle. The wound was closed a nearly as possible with adhesive plasters: the flap was turned back, dressed with simple cerate, enveloped in cotton, the whole being secured by a few light turns of a roller. In six days about one-fourth of the flap had sloughed; the sloughing being arrested by yeast poultices. At the end of two weeks the flap by sloughing and contraction had dimished to about half its original size. It was however three times its original thickness and vascular; the thick cuticle had fallen off, and left a soft, pliant skin, and the edges were cicatrized.

On the 12th of April, having placed upon his head a cap furnished with straps, the flap and the nose were made raw, and by means of a sling to which the straps from the cap were attached, the arm and hand were brought up, and the skin secured to the nose by five small sutures. A pillow was slung under the arm-pit, against which the elbow rested. Two students now remained in constant attendance, being relieved every four or six hours by others. The patient was kept in this position seventy-two hours, when union having taken place, the flap was separated from the hand. Of that which remained attached to the face, a small portion sloughed, but suffincient remained to nearly complete this ala.

The patient now returned home, and in October, 1848, he again placed himself under my care in the "Buffalo Hospital of the Sisters of Charity."

*Seventh* operation. The left ala was not complete, and on the — of Oct. I raised by careful dissection, with instruments adapted to the purpose, a thick plate covered with mucous membrane from *within* the nostril, leaving a pedicle downward and outward at that part of the opening of the nostril which is nearest the external commissure of the mouth: this plate I brought out, and raising it, I attached it to the apex of the nose and to the lower border of the ala.

This operation, which is entirely original with me, I was enabled to make from the fact that the burn had destroyed the roots of most of the hairs within the nostril, and had also left the mucous membrane and the subjacent tissue much thickened. The success has exceeeed my expectations.

## Figures and Tables

**Figure f1:**
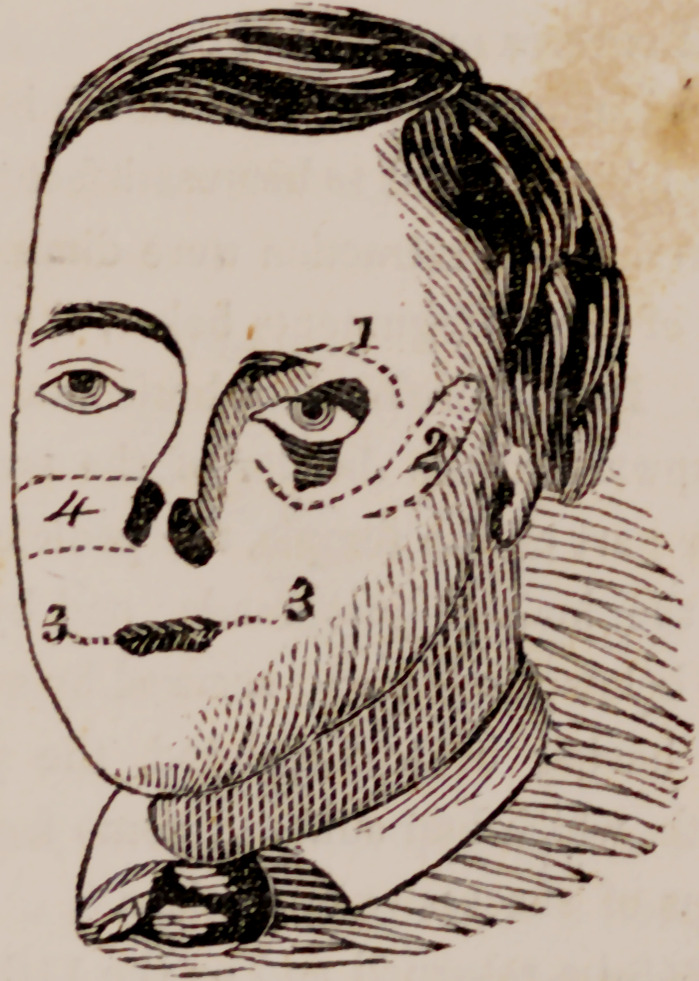


**Figure f2:**